# Conservative Management of Interstitial Pregnancy With Beta-hCG of Over 39,000 mIU/mL: A Case Report

**DOI:** 10.7759/cureus.26027

**Published:** 2022-06-17

**Authors:** Tom J Mayuga, Amir Antonios, Shanna Hutchins, Hedwige Saint-Louis

**Affiliations:** 1 Family Medicine, WellStar Atlanta Medical Center, Atlanta, USA; 2 Family Medicine, Ross University School of Medicine, Atlanta, USA; 3 Obstetrics and Gynecology, WellStar Atlanta Medical Center, Atlanta, USA

**Keywords:** methotrexate, beta-hcg, hemorrhagic shock, ectopic pregnancy, interstitial ectopic pregnancy

## Abstract

Interstitial ectopic pregnancy can be a life-threatening condition as the myometrial tissue around the gestational sac is thin. Furthermore, the interstitial aspect of the fallopian tubes is highly vascularized. Thus, a rupture in this area can result in catastrophic hemorrhage, hemoperitoneum, and shock. Therefore, surgical management is often the preferred mode of therapy. This report identifies the successful medical management of a patient with interstitial ectopic pregnancy with β-hCG of more than 39,000 utilizing methotrexate.

## Introduction

Ectopic pregnancy is defined as pregnancy occurring outside of the uterine cavity and accounts for 2% of all reported pregnancies [1,2]. The majority of ectopic pregnancies form within the fallopian tubes [1]. One rare form of ectopic pregnancy is called interstitial pregnancy which forms within the proximal and intramural portion of the fallopian tube, usually at the junction of the fallopian tube and uterine cavity. Interstitial pregnancies account for approximately 2% of ectopic pregnancies [3]. Most ectopic pregnancies are caught early and can be managed appropriately with medical therapies. However, in unstable patients, prompt surgical intervention is necessary. Ruptured ectopic pregnancies account for 2.7% of all pregnancy-related deaths due to hemorrhagic shock.

Ectopic pregnancies are diagnosed via transvaginal ultrasound and β-hCG levels. Whereas the designation of interstitial ectopic pregnancy is based on image findings alone, either transvaginal ultrasound or less commonly, MRI. A normal intrauterine pregnancy is generally visible on ultrasound at β-hCG levels of 1,500-2,000 mIU/mL. Medical treatment for interstitial pregnancy involves termination of the pregnancy with either single or multiple doses of methotrexate. Hemodynamically stable patients with an unruptured mass, and without contraindications to methotrexate use, can undergo medical management. In the context of this case some important relative contraindications to methotrexate use are a high initial β-hCG concentration, and an ectopic pregnancy mass greater than 4 cm [1]. A β-hCG level >5,000 mIU/mL has traditionally been used as a cut-off for medical management due to higher treatment failure rates above this level [4]. Some studies recommend using an initial β-hCG level >2,000 mIU/mL as a cut-off [5]. Multiple surgical approaches exist for the management of interstitial pregnancies but generally involve resection of the uterine cornua or hysterectomy [6]. Surgical intervention is indicated in hemodynamically unstable patients, when there are signs of intraperitoneal bleeding, or in suspicion of a ruptured mass. The surgical approach can cause varying degrees of complications most notably future difficulty with fertility.

Limited data are available highlighting the success of medical management in ectopic pregnancies with an initial β-hCG above 5,000 mIU/mL since this value stands as a cut-off point for many clinicians and clinical trials. A better understanding of treatment failure rate in later stages of pregnancy would help guide clinicians and patients in choosing treatment options and limiting the complications of the surgical approach. This report aims to elaborate on the use of medical management for interstitial pregnancies with initial β-hCG levels greater than 5,000 mIU/mL leading to a successful clinical outcome. This article was previously presented to the clinical research committee of the American College of Osteopathic Family Physicians on February 24, 2022.

## Case presentation

**Figure 1:**
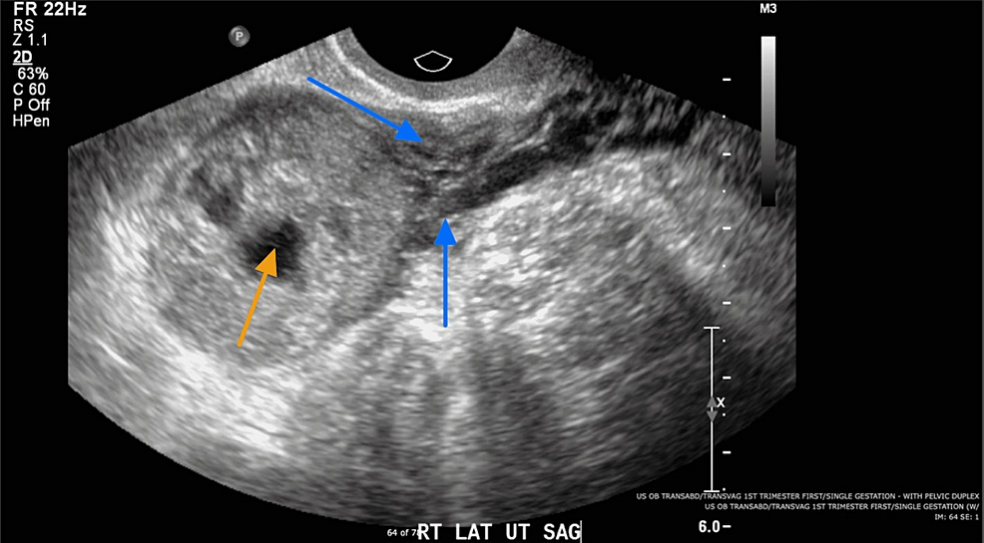
Transvaginal ultrasound indicating echogenic structure in the right lateral aspect of the uterine fundus. The central cystic component is indicated with the orange arrow. Blue arrows highlight a hypo-echoic myometrial band between the endometrial echos and the gestational sac.

**Figure 2:**
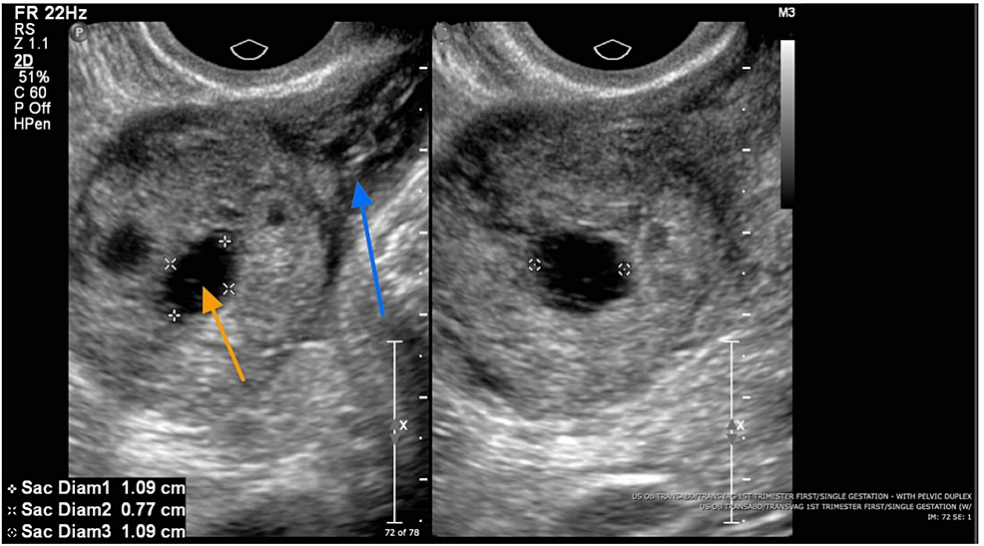
Along the right lateral aspect of the uterine fundus there is a thick-walled round structure measuring 3.5 x 3.3 x 3.5 cm as seen in this enhanced image. The central cystic component measures 1.1 x 1.1 x 0.8 cm indicated by the orange arrow. No definite yolk sac or embryo is identified. The blue arrow denotes the hypo-echoic band of myometrium between the endometrial echos and gestational sac.

**Figure 3:**
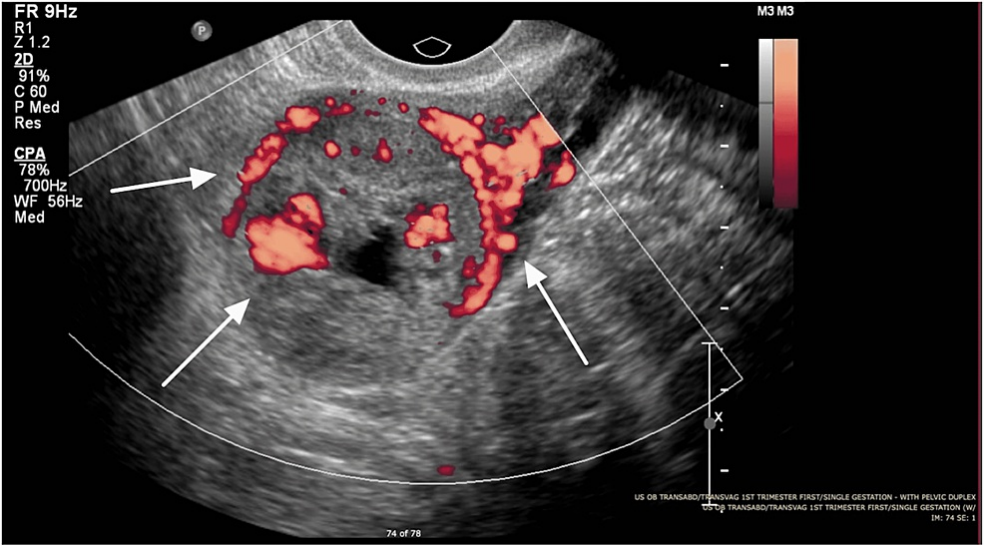
Doppler ultrasound of interstitial ectopic pregnancy. There is peripheral and internal flow on color Doppler indicated by the white arrows in a characteristic “ring of fire pattern.”

The patient is a 30-year-old gravida 1 para 0 female with no relevant medical history who presented to the ER with crampy abdominal pain and nausea. The pain was described as waxing and waning in nature. She denied any vaginal bleeding, dysuria, vomiting, diarrhea, or constipation. The patient’s vital signs were unremarkable in that she was hemodynamically stable and afebrile, and a physical exam revealed only lower abdominal tenderness to palpation. Urine pregnancy testing was positive revealing a β-hCG quant of >39,000 mIU/mL. The patient’s hemoglobin was 10.4 and hematocrit was 30.5. A first trimester US was performed, which revealed a thick-walled round structure measuring 3.5 x 3.3 x 3.5 cm. The central cystic component measured 1.1 x 1.1 x 0.8 cm. No definite yolk sac or embryo was identified (Figures 1-3).

After extensive counseling regarding the risks versus benefits of both medical and surgical management, the patient chose to proceed with medical intervention as she was concerned with the impact surgical management could pose to her ability to bear children in the future. The patient received an initial dose of methotrexate 50 mg/m^2^ intramuscularly with a second 50 mg/m^2^ dose approximately 24 hours later. She was monitored for adverse effects to the medication then subsequently discharged under stable condition following the second dose with instructions to follow up to the ED within 3-4 days for a repeat ultrasound and β-hCG level. On the first follow up, three days after hospital discharge, β-hCG was noted as down trending from initial presentation with a value of 28,000 mIU/mL. However, ultrasound revealed a mild increase in size of the ectopic pregnancy since initial examination, now noted as 4.5 x 3.5 x 3.4 cm. Additionally, the patient endorsed occasional lower abdominal cramping, so as a precaution, she was subsequently admitted for observation. She remained hemodynamically stable throughout the evening and her pain resolved, so she was discharged the following morning. On the day of discharge, β-hCG had decreased to 21,125 mIU/mL. Repeat US was reassuring and showed a complex right adnexal lesion now measuring 3.4 x 2.9 x 3 cm an overall reduction from even her initial presentation. The patient was counseled on the continued need for serial β-hCG levels, and instructed to follow up with a laboratory of her choosing every two weeks for blood draws to ensure the β-hCG level continued to down trend. Overall, the patient underwent six laboratory follow ups for β-hCG level assessment, which she completed over the next three months. The quantitative β-hCG level from her first outpatient laboratory visit was noted as 4,292 mlU/mL, which decreased to 2,956 on the second visit, 321 by the third visit, 236 by the fourth visit, and 118 by the fifth visit. The patient was noted to have a β-hCG of 49 on her sixth and final outpatient blood draw.

## Discussion

Uterine anomalies, pelvic inflammatory disease (PID), previous ectopic pregnancy, and in vitro fertilization are just some of the risk factors for interstitial ectopic pregnancy [7]. The patient in this case report does not have any known risk factors. However, natural causes may also play a role in interstitial implantation. Due to the vascularity of the myometrium, interstitial pregnancy is associated with an increased risk for hemorrhagic shock and hemoperitoneum compared to other forms of ectopic pregnancy [8]. Additionally, interstitial pregnancy is associated with higher rates of maternal mortality [7]. In the event of ruptured interstitial pregnancy with hypovolemic shock, an emergency laparotomy and cornual resection or hysterectomy would likely be necessary [9]. For this reason, it is often managed surgically. However, in hemodynamically stable patients, medical management can be attempted.

A report by Tulandi et al. details the surgical and medical management of 32 cases of interstitial ectopic pregnancy [10]. Of those cases, eight were treated with methotrexate (MTX). Four of these women received MTX systemically while the other four received the medication locally; two via US-guided infiltration and two laparoscopically. Three out of the four patients who received MTX systemically failed medical management [10]. In contrast, a prospective observational study by Jermy et al. examined conservative management of interstitial pregnancy in 20 patients [11]. In the study, the patients had an average β-hCG of 6,452. Sixteen of the 17 patients who received systemic MTX were treated successfully [11]. While the success rate of this study is encouraging, medical management with MTX is thought to have an overall success rate of approximately 35% [9,10,12].

Many obstetric physicians would proceed with an invasive approach in patients with interstitial pregnancy and a significantly elevated β-hCG as described in this case, as it is the definitive treatment and often the approach with the potential for fewer complications. However, due to fertility concerns, our patient opted for conservative management despite extensive education about risk vs benefits. Overall, the follow-up required her to complete seven additional visits, six of which were performed at an outpatient laboratory and spanned a total of three months. While the time commitment for this approach was acceptable for our patient considering she was adamantly opposed to operative intervention, this may not be the case for everyone. A myriad of factors can influence a patient's decision regarding surgical versus medical management of interstitial ectopic pregnancy. For instance, the fastidious nature of follow-up required for medical management could be unrealistic for those without reliable transport or those without continued leave from employment for serial blood draws. Conversely, patients with limited means may see the cheaper cost of outpatient laboratory follow-up more alluring than a costly operation. In a retrospective review comparing the cost of single dose methotrexate to surgical intervention, Stovall et al. noted that patients undergoing surgical intervention for ectopic pregnancy incurred 4.8 times more charges than individuals managed with methotrexate [13]. Additionally, the administration of methotrexate is non-invasive and can be performed at the outpatient level. Whereas surgical interventions, even minimally invasive, laparoscopic procedures, still require the administration of anesthesia and a subsequent recovery period for the patient. While management of an interstitial ectopic pregnancy largely depends on the underlying medical status of the patient at initial presentation, socioeconomic factors and patient preference must be taken into consideration as well. This case demonstrated that interstitial pregnancy with a β-hCG as high as 39,000 mIU/mL can be managed medically if the patient is hemodynamically stable without signs of acute abdomen or intraabdominal hemorrhage on ultrasound. However, this approach requires that the patient is able and will reliably follow up for serial β-hCGs. Fastidious follow up should continue until the β-hCG value is 0.

## Conclusions

Clinicians must have a low threshold for operative treatment by laparoscopy or laparotomy as rupture of interstitial pregnancy can quickly lead to hemorrhagic shock and death. However, this case showed that medical management of interstitial pregnancy with methotrexate with a β-hCG value as high as 39,000 mIU/mL is a viable option. For consideration of medical management, patients must be anatomically and hemodynamically stable throughout the course of medical treatment with the knowledge that surgical intervention may become necessary if the patient becomes hemodynamically unstable. Additionally, the patient must receive appropriate discharge education regarding the importance of close follow-up and return precautions prior to implementing this treatment modality.
